# Revisiting the (anti)inflammatory effects of prolonged fasting: the importance of baseline-dependent responses

**DOI:** 10.3389/fnut.2025.1715341

**Published:** 2025-11-14

**Authors:** Robin Mesnage, Franziska Grundler, Françoise Wilhelmi de Toledo

**Affiliations:** 1 Buchinger Wilhelmi Clinic, Überlingen, Germany; 2 Department of Nutritional Sciences, Faculty of Life Sciences and Medicine, School of Life Course Sciences, King's College London, London, United Kingdom

**Keywords:** fasting, inflammation, CRP, immune response, hormesis

## 1 Introduction

Fasting has long been recognized for its anti-inflammatory potential (1). Clinical and observational studies have shown that prolonged fasting can alleviate symptoms in chronic inflammatory disorders, with rheumatoid arthritis being one of the best-documented examples (2). Patients undergoing fasting interventions frequently report reductions in joint pain and stiffness, accompanied by measurable decreases in disease activity scores. These findings, together with evidence of improved outcomes in other inflammatory and autoimmune conditions, have contributed to the perception of fasting as an inherently anti-inflammatory therapy.

However, the evidence from biomarker studies does not always align with the reported clinical improvements. Several controlled trials have documented increases in circulating inflammatory markers such as C-reactive protein (CRP) during prolonged fasting (3). Such increases could be interpreted as suggestions that fasting provoke a pro-inflammatory response, raising questions about how these short-term dynamics reconcile with longer-term clinical benefits.

In this article, we argue that baseline inflammatory status offers a unifying framework to reconcile these apparently contradictory findings. We propose that fasting elicits a biphasic, baseline-dependent immune response: a transient adaptive increase in inflammatory activity in some individuals, followed by longer-term normalization or resolution in those with elevated baseline levels. Recognizing this dynamic is essential for interpreting fasting research and guiding its clinical application in a safe and personalized manner.

## 2 Evidence for baseline-dependent responses in a large cohort of 1,422 individuals

We previously published health effects of prolonged fasting in an observational cohort of 1,422 participants who volunteered for a 4–21-day Buchinger fasting protocol (4). Blood examinations before and at the end of the fasting period. In this first analysis published in 2019, we did not stratify patients according to baseline-dependent responses and focused on the contribution of sex and fasting duration. We reported that mean CRP concentrations significantly increased from 2.85 ± 0.14 mg/L before fasting to 4.30 ± 0.20 mg/L during fasting in the whole cohort, while providing caution on the clinical implications of this effects. This data was recently analyzed by Commissati et al. and used as a support of their conclusions that prolonged fasting triggers inflammation potentially impacting cardiometabolic health (5).

**Figure 1:**
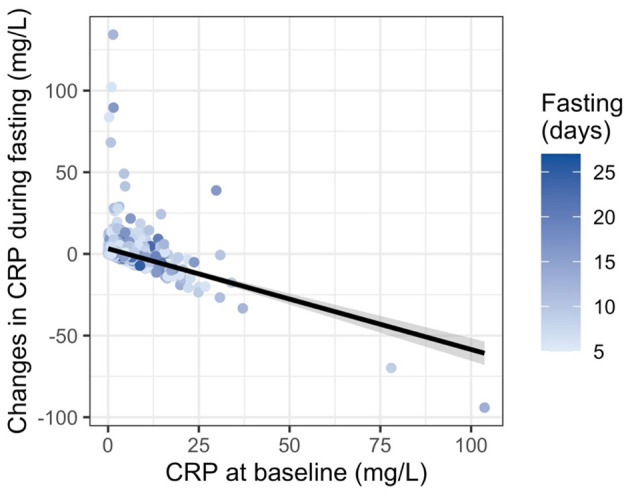
Relationship between baseline CRP levels and changes in CRP during prolonged fasting in 1,422 individuals. Each point represents one individual, with the *x*-axis showing CRP concentration at baseline and the *y*-axis indicating the absolute change in CRP by the end of fasting. Point color reflects the duration of the fasting period in days, with darker shades indicating longer fasts. A linear regression line with 95% confidence interval illustrates the overall trend. Individuals with elevated baseline CRP tended to experience a greater reduction in CRP, suggesting a normalization of inflammation during fasting.

While a consistent increase in CRP during fasting is supported at the group level, changes in CRP during fasting varied strongly with baseline values. To characterize these baseline-dependent responses, participants were stratified by baseline CRP into low (< 1 mg/L), moderate (1–10 mg/L), and elevated (>10 mg/L) groups. In the elevated CRP group (*n* = 90), mean CRP decreased from 17.3 to 10.9 mg/L. Participants with moderate baseline CRP (*n* = 636) showed an increase from 3.53 to 5.93 mg/L, while those with low baseline CRP (*n* = 694) exhibited an increase from 0.36 to 1.96 mg/L. Participants with elevated CRP at baseline thus experienced more significant reductions, whereas those starting with low CRP more often showed modest increases (Figure 1). An ANCOVA with change in CRP as the dependent variable and baseline CRP as a covariate confirmed a significant negative association between baseline CRP and CRP change (*p* < 0.001). This suggests a “normalization” phenomenon, where fasting modulates inflammation toward a healthier equilibrium depending on initial status.

## 3 Discussion and clinical implications

The rise in inflammatory markers observed during fasting should be distinguished from chronic low-grade inflammation seen in metabolic or autoimmune disorders. In contrast, our clinical experience as well as multiple clinical studies have shown that prolonged fasting can reduce disease activity in chronic inflammatory conditions like rheumatoid arthritis (2). These benefits have been consistently reported despite occasional short-term increases in acute-phase proteins such as CRP.

Our analysis provides a mechanistic rationale for this apparent discrepancy. The negative association between baseline CRP and its change during fasting indicates that individuals with higher baseline inflammation experience reductions, whereas those with low baseline CRP display small increases. This pattern suggests that fasting triggers a biphasic, adaptive immune modulation: an initial stress-related activation followed by immune recalibration toward homeostasis. Such a mechanism aligns with the concept of hormetic stress, in which transient perturbations stimulate systemic resilience.

Limitations should be acknowledged. A small number of participants (*n* = 7) with low baseline CRP (< 1 mg/L) showed disproportionately large increases in CRP during fasting (>10 mg/L). The available data do not provide information on possible causes such as intercurrent infection or other inflammatory processes. Although these outliers contribute to the variability observed in the low-CRP group, their inclusion does not affect the overall negative association between baseline CRP and fasting-induced change. It should also be noted that CRP was measured only before and after fasting in this cohort; thus, the temporal pattern of change cannot be directly confirmed. In this regard, previous work has shown that cytokine levels measured during food reintroduction display a transient inflammatory activation consistent with a reactivation of postprandial immune responses (6).

Recognizing this baseline dependence has direct clinical implications. Increases in CRP during fasting should not necessarily be interpreted as deleterious, particularly in individuals with low baseline inflammation, especially if they are asymptomatic and remain within the norm range. Rather, these shifts may represent a physiological resetting process that precedes improved immune regulation. Conversely, individuals with elevated inflammatory activity appear to benefit through normalization of CRP levels and clinical symptom relief.

We therefore recommend that future analyses stratify participants by baseline inflammatory and metabolic risk factors. This would provide a more nuanced understanding of the physiological responses to prolonged fasting and help clarify for which populations these interventions may be most beneficial.
